# Harnessing mRNA for heart health: a new era in cardiovascular treatment

**DOI:** 10.7150/thno.111503

**Published:** 2025-07-02

**Authors:** Fengli Peng, Zhimei Qiu, Zimu Wang, Fuhai Li, Fating Zhou, Shuai Yuan, Jinsong Yuan, Yan Wang, Yongchao Zhao, Chaofu Li, Bei Shi

**Affiliations:** 1 Department of Cardiology, Affiliated Hospital of Zunyi Medical University, Zunyi, China.; 2 Department of Cardiology, Zhongshan Hospital, Fudan University, Shanghai Institute of Cardiovascular Diseases, Shanghai, China.; 3 Department of Cardiology, Affiliated Hospital of Qingdao University, Qingdao, China.; 4 Department of cardiology, Chongqing University Central Hospital (Chongqing Emergency Medical Center), College of Bioengineering, Chongqing University, Chongqing, China/

**Keywords:** mRNA-based drug formulations, gene therapy, novel therapies, nanomedicine, cardiovascular diseases

## Abstract

mRNA serves as a versatile platform for the expression of paracrine factors, thereby promoting cardioprotection and regeneration. In recent years, mRNA and gene editing technologies have emerged as innovative tools for tackling complex diseases. Among these, mRNA therapeutics offer distinct advantages, including favorable immunological properties, strong safety profiles, and superior flexibility compared to conventional gene-based vaccines. Specifically, they can elicit a balanced immune response—encompassing both cellular and humoral immunity—without being limited by MHC haplotypes. Furthermore, mRNA therapy represents a particularly safe treatment modality. As a minimal and transient carrier of genetic information that does not integrate into the host genome, it significantly reduces the risk of insertional mutagenesis. Importantly, mRNA can be used to express virtually any protein without requiring changes to the production process, thus offering substantial flexibility in drug development. Collectively, these attributes highlight the great potential of mRNA as a next-generation therapeutic platform, particularly for cardiovascular diseases. This review summarizes recent progress in the development and application of mRNA-based drug formulations for cardiovascular therapy.

## Introduction

Cardiovascular disease (CVD) remains a leading global health burden, contributing substantially to both disability and mortality. According to the World Health Organization, CVD is responsible for approximately 18 million deaths annually, representing nearly one-third of all global fatalities. However, the impact of CVD extends beyond mortality 1. It also leads to long-term disability and escalating healthcare costs, which in turn significantly diminish the quality of life for patients and their families. Although various treatment options are available—including antihypertensive medications, lipid-lowering agents, and cardiac surgical interventions—several critical challenges remain. These include delayed diagnosis, poor patient adherence, unequal access to healthcare, and a lack of innovative therapies. Collectively, these factors hinder the effective prevention, treatment, and long-term management of cardiovascular disease 2.

To address these limitations, emerging therapies—including cell-based treatments and nucleic acid therapeutics—aim to target the root causes of disease, enabling more precise and effective interventions. These strategies hold great potential for meeting unmet clinical needs, improving patient outcomes, and potentially altering the course of cardiovascular disease. Among these innovations, mRNA has emerged as a particularly versatile platform for both drug and vaccine development. It offers exceptional flexibility in molecular design, scalable production, and rapid adaptation to diverse clinical applications. By encoding virtually any protein, mRNA enables the development of both prophylactic and therapeutic vaccines for a wide range of diseases, including infections, cancers, and protein deficiency disorders. In the context of cardiovascular disease, mRNA-based therapies show promise in enhancing cardiac repair, reducing the incidence of adverse cardiovascular events, and improving patients' quality of life 3. Furthermore, mRNA technology facilitates the advancement of personalized medicine by enabling therapies to be tailored to individual genetic and clinical profiles, thereby increasing the precision and effectiveness of treatment strategies 4.

**Figure 1:**
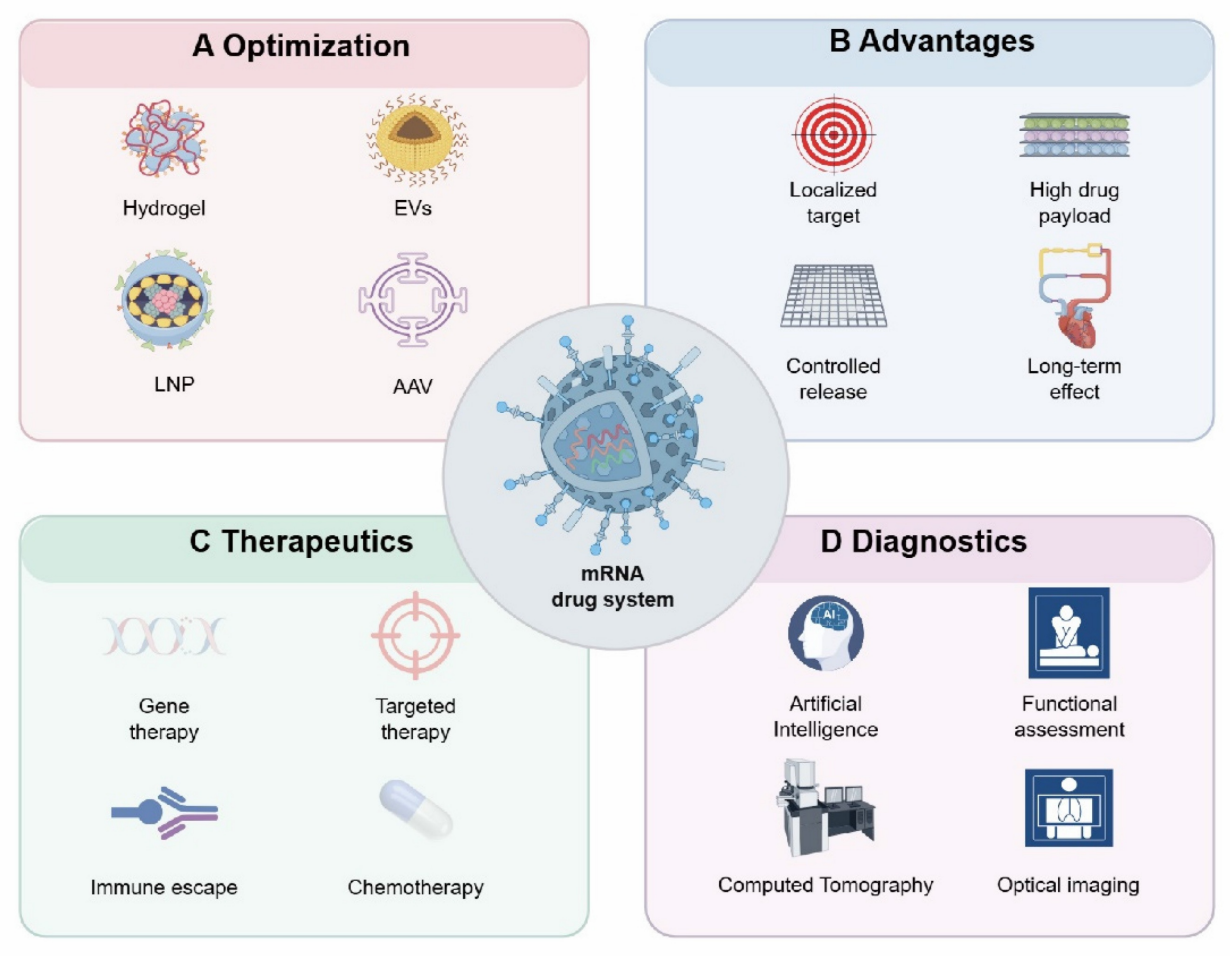
** Design and application of mRNA drug system (by Figdraw).** After mRNA is modified with different drug carriers, it can be targeted to the injured heart for repair, and this design mode can be used as a diagnostic medicine platform for the centralized diagnosis of the cardiovascular system. A: carrier of mRNA drugs, such as AAV, LNP, hydrogel, EVs, etc.; B: Advantages of mRNA technology, such as: targeting heart, high drug load, controlled release and long-acting; C: mRNA technology can realize gene targeting therapy and improve cardiac function through immune escape; D: mRNA technology can realize the diagnosis of cardiovascular system. Abbreviations: EVs, Extracellular vesicles; LNP, Lipid nanoparticles; AAV, Adeno-associated virus.

Building on this foundation, this narrative review aims to provide a comprehensive overview of emerging therapeutic strategies for cardiovascular disease, with a particular focus on the development of mRNA-based drug formulations. It outlines key considerations such as mRNA manufacturing and quality control, structural design and formulation strategies, antigen and protein expression, and the immunological characteristics of mRNA. The review also highlights the therapeutic potential of mRNA in cardiovascular applications. Furthermore, it discusses the challenges associated with the clinical adoption of these therapies, including regulatory, economic, and ethical factors, and explores future directions for their implementation. Ultimately, this review seeks to contribute to global efforts aimed at reducing the burden of cardiovascular disease and improving cardiovascular health outcomes** (Figure 1)**.

## 1. Overview of mRNA Drug Formulations

**Figure 2:**
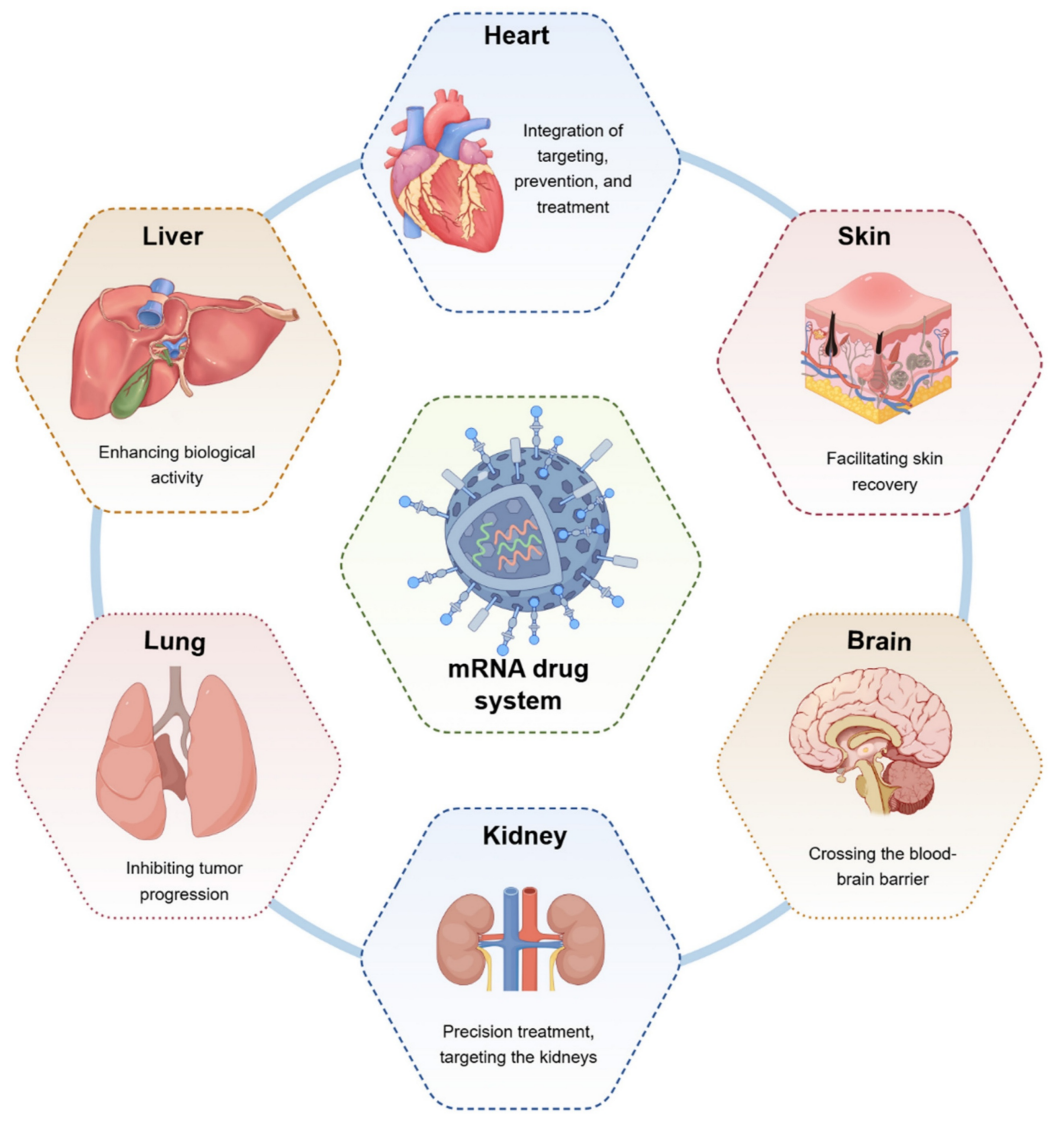
** Therapeutic role of mRNA technology in different diseases (by Figdraw).** mRNA technology can exert different biological effects on the heart, liver, lungs, kidneys, brain, skin, and other organs to facilitate disease repair.

**Figure 3:**
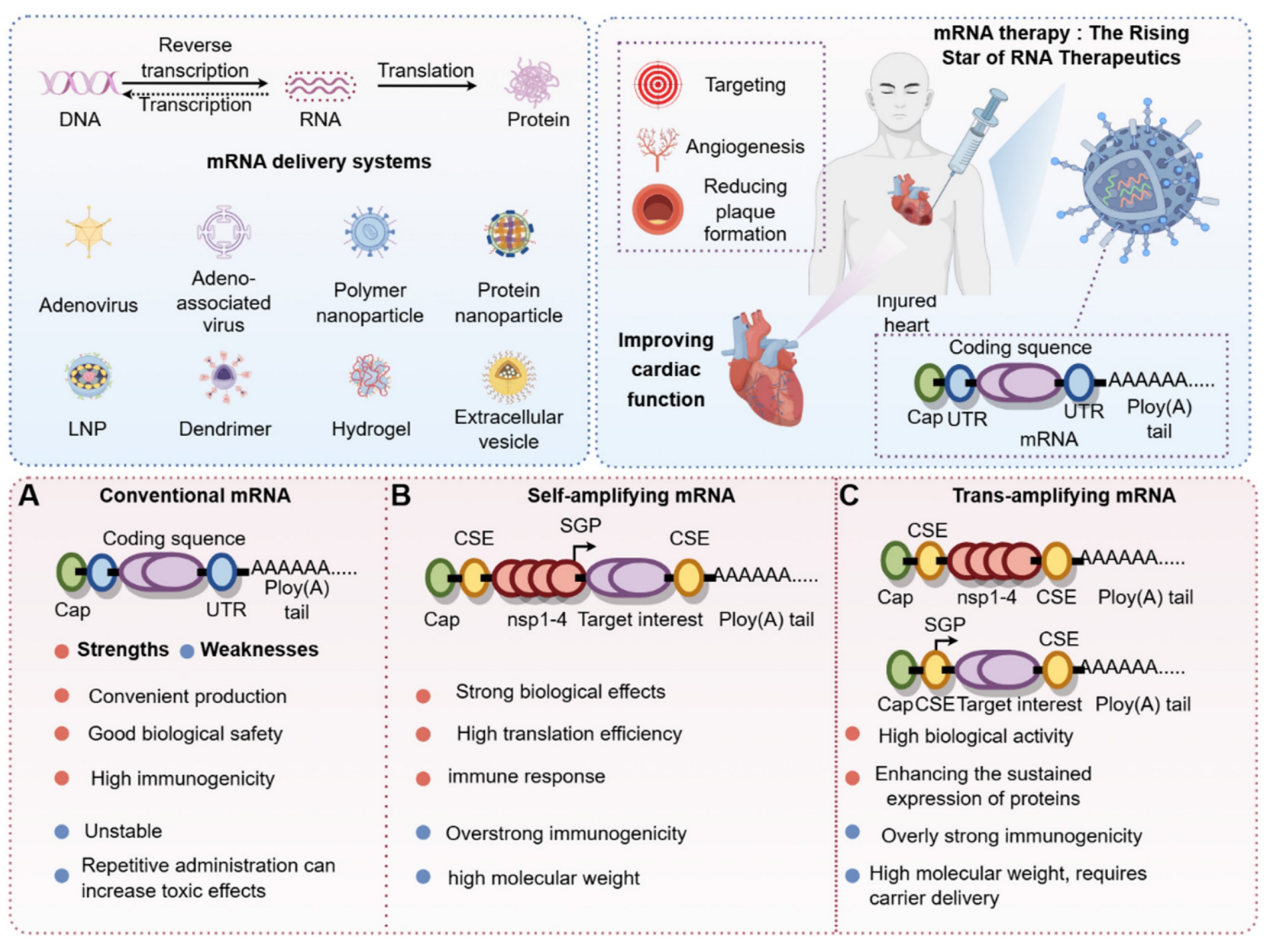
** Classification of mRNA and timeline of exploration (by Figdraw).** According to the central dogma, DNA is transcribed into RNA, which is then translated into protein. mRNA plays a crucial role in the RNA translation process. Through chemical modifications or carrier-mediated modifications, the stability of mRNA can be significantly enhanced to maximize its biological effects. Additionally, depending on mRNA classifications—such as conventional mRNA, self-amplifying mRNA, and trans-amplifying mRNA—we have systematically compared the advantages and disadvantages of these distinct mRNA categories. Abbreviations:DNA, Deoxyribonucleic acid; RNA, Ribonucleic acid; LNP, Lipid nanoparticles; UTR, Untranslated region.

mRNA-based nucleic acid therapeutics were first proposed three decades ago as a novel strategy for developing vaccines that are safe, versatile, and easy to manufacture **(Figure 2)**. The basic structure of an mRNA molecule comprises five key components: a 5′ cap, a 5′ untranslated region (UTR), an open reading frame (ORF) encoding the target protein, a 3′ UTR, and a poly (A) tail. Unlike traditional vaccines, mRNA does not integrate into the host genome, thereby eliminating the risk of insertional mutagenesis. Additionally, mRNA vaccines can be produced using cell-free systems, allowing for rapid, scalable, and cost-efficient manufacturing 5. A notable advantage of mRNA technology is its ability to encode multiple antigens within a single formulation, which enhances immune responses against rapidly evolving pathogens and provides broad-spectrum protection against various microorganisms and viruses 6 **(Figure 3)**.

**Figure 4:**
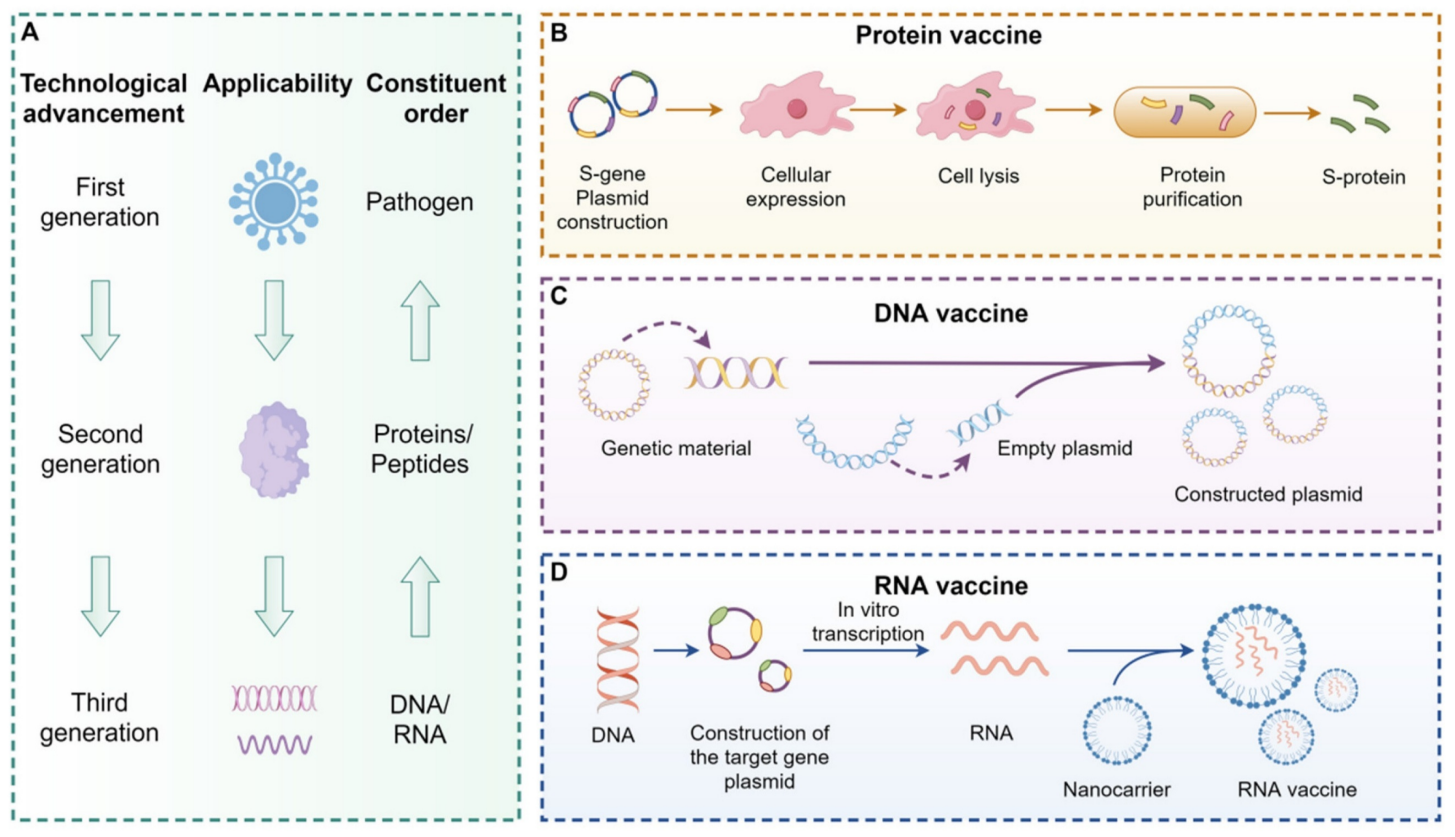
** Vaccine development (by Figdraw).** Vaccines can activate human immune cells, enabling them to recognize specific viral protein structures, thereby achieving an immune response. Vaccines are categorized into three generations: the first generation is based on pathogens (A), the second generation is based on proteins or peptides (B), and the third generation is based on DNA (C) or RNA (D). Compared to protein vaccines, mRNA vaccines eliminate the step of culturing the antigen *in vitro*, significantly reducing the production cycle and accelerating the development speed. Moreover, compared to DNA vaccines, mRNA vaccines are safer because they do not involve fragment insertion. Abbreviations:DNA, Deoxyribonucleic acid; RNA, Ribonucleic acid.

Building on this foundation, vaccine development from a biological standpoint encompasses multiple strategies, including DNA vaccines, mRNA vaccines, and protein-based vaccines 7, as illustrated in **Figure 4**. The core mechanism of vaccination is to activate immune cells so they can recognize specific viral protein structures and initiate an immune response 5. Among these approaches, mRNA vaccines offer several notable advantages. Compared to protein-based vaccines, they eliminate the need for *in vitro* antigen production, thereby significantly reducing manufacturing timelines. In contrast to DNA vaccines, mRNA does not require nuclear entry, which minimizes the risk of reverse transcription and integration into the host genome 8. These properties position mRNA vaccines as a safer and more promising platform for infectious disease prevention.

Despite these advantages, translating mRNA into a viable therapeutic modality for both rare and common diseases remain a significant challenge 9, 10. Major obstacles include the need for high levels of protein expression, poor tissue bioavailability, rapid degradation in circulation, and inefficient targeted delivery. Moreover, repeated administration can trigger innate immune responses, leading to diminished protein expression over time. Addressing these limitations underscores the urgent need to accelerate the research and development of mRNA-based therapeutics in the current era of biomedical innovation.

## 2. Design and Optimization Strategies for mRNA Drug Formulations

### 2.1 Design strategies for mRNA drug formulations

**Figure 5:**
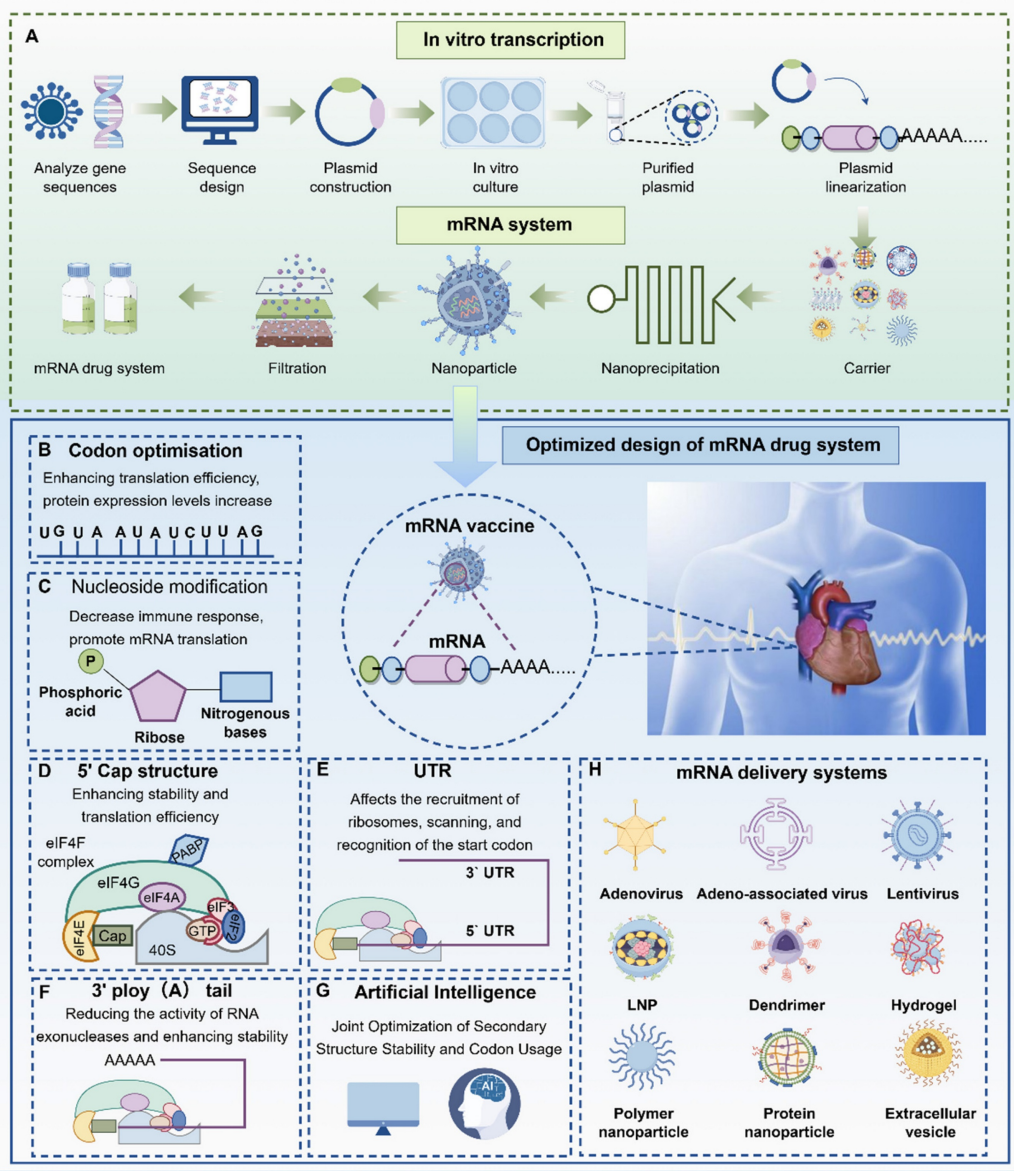
** Design and optimization strategies for mRNA vaccines (by Figdraw).** mRNA vaccines are produced by selecting gene sequences, plasmid construction, *in vitro* culture, and optimizing mRNA (A). mRNA design mainly includes: codon optimization (B), nucleic acid design (C), 5' cap design (D), UTR design (E), 3' A-tail design (F), artificial intelligence algorithm design (G), and mRNA vector optimization strategy (H). Abbreviations: DNA, Deoxyribonucleic acid; RNA, Ribonucleic acid; LNP, Lipid nanoparticles; UTR, Untranslated region.

Key attributes such as vaccine efficacy and thermostability are largely determined by the intrinsic properties of the mRNA molecule 11. To enhance these properties, researchers around the world are actively working to improve the translational efficiency and stability of mRNA. This is especially important in ischemic cardiac tissue, where improved translation can reduce the amount of mRNA required per dose and enable higher, more sustained protein expression following a single administration. These improvements can be achieved by modifying specific structural elements of the mRNA molecule, including the 5′ and 3′ untranslated regions (UTRs), the poly (A) tail, codon optimization, and chemical modification of nucleotides** (Figure 5)**.

#### 2.1.1 Optimization of coding sequences

Optimizing codon usage can enhance translational efficiency and reduce the formation of secondary structures that hinder translation 12. However, in humans, codon usage bias is not closely correlated with tRNA abundance or gene expression, limiting the effectiveness of codon optimization—especially when the open reading frame (ORF) is derived from human or other mammalian sequences. To further improve translation, the start codon should be placed within a Kozak consensus sequence, and the sequence surrounding the stop codon can also be optimized. Additionally, upstream start codons that precede the correct initiation site should be removed to avoid aberrant translation initiation 13. Ultimately, refining the coding sequence and minimizing innate immune activation through rational vector design can significantly improve both mRNA stability and protein expression.

#### 2.1.2 Nucleotide modification

Nucleotide modification is a critical component in the development of mRNA therapeutics. Unmodified nucleotides can be recognized by intracellular RNA sensors, triggering innate immune responses that lead to rapid mRNA degradation and inhibition of protein translation 14. In 2005, Katalin and colleagues 15 demonstrated that specific uridine modifications—such as m5C, m5U, and pseudouridine (Ψ)—can suppress the activation of certain human Toll-like receptors (TLRs), whereas unmodified RNA activates multiple TLRs, including TLR3, TLR7, and TLR. To investigate the effects of nucleotide modifications, researchers have utilized cardiac cells and tissues to assess immunogenicity, plasma stability in mice, and protein expression efficiency. Compared to unmodified mRNA, all modified mRNAs significantly reduced the activation of key innate immune genes, such as IFN-α, IFN-β, and RIG-I. They also demonstrated enhanced stability, greater RNA integrity in mouse plasma, and prolonged persistence. Moreover, modified mRNAs improved protein translation, exhibited extended *in vitro* expression kinetics, and showed favorable *in vivo* pharmacokinetic profiles. These features support efficient, localized, and rapid gene delivery—achieving transient expression within minutes and sustained expression for several days in adult cardiac tissue. The platform is non-immunogenic and demonstrates favorable biodistribution, with more than 20% of the administered mRNA localizing in the left ventricle. Collectively, these findings have had a significant impact on mRNA vaccine design and have further advanced the therapeutic application of mRNA.

#### 2.1.3 5′cap design

The addition of a 5′ cap to mRNA significantly enhances translational efficiency by promoting recognition by eukaryotic initiation factor 4E (eIF4E), thereby improving mRNA stability and facilitating effective protein synthesis 16, 17. Capping is typically performed during *in vitro* transcription (IVT) and can be implemented either co-transcriptionally (one-step) or post-transcriptionally (two-step). However, incorrect capping—especially in the reverse orientation—can lead to rapid mRNA degradation and substantially reduced translation efficiency.

#### 2.1.4 3′poly-A tail

The poly(A) tail enhances mRNA stability by inhibiting ribonuclease activity and binding to poly(A)-binding protein (PABP), which subsequently recruits eIF4G and eIF4E. These interactions increase the affinity for the 5′ cap and promote the formation of a circular mRNA structure, thereby facilitating efficient translation 18, 19. Despite its importance, the optimal length of the poly(A) tail has not been clearly defined. Therefore, determining the appropriate tail length is essential for maximizing both mRNA stability and translational efficiency.

#### 2.1.5 Regulation by untranslated regions (UTRs)

The selection of untranslated regions (UTRs) plays a critical role in regulating mRNA stability and translational efficiency 20. For instance 21, the combination of the β-globin 5′-UTR and α-globin 3′-UTR has been shown to enhance translation in a metastatic melanoma vaccine model. However, certain UTR features—such as upstream start codons and stable secondary structures near the 5′ end—can interfere with ribosome recruitment and start codon recognition, thereby impairing translation initiation 22, 23. These elements should be carefully avoided during mRNA design. A recent study 24 investigated transcriptomic and proteomic changes in the left ventricle of mice at 4 and 24 hours following myocardial infarction (MI), compared with sham-operated controls. Several differentially expressed genes with distinct 5′-UTR sequences were identified. Among them, the 5′-UTR of the carboxylesterase 1D (Ces1d) gene—associated with fatty acid metabolism—was found to double the translation of luciferase (Luc) mRNA modified with N1-methyl-pseudouridine (m1Ψ) in infarcted cardiac tissue. Incorporating the Ces1d 5′-UTR into Luc or GFP reporter mRNAs resulted in both increased and prolonged protein expression. Notably, an RNA element within the Ces1d 5′-UTR, termed "Element D," enhanced translation by 2.5-fold compared with synthetic 5′-UTRs under MI conditions 24. In a parallel development, Hadas *et al.* 25 established an improved *in vitro* mRNA synthesis protocol that yielded higher mRNA output. By optimizing the ratio of anti-reverse cap analog (ARCA) to N1-methyl-pseudouridine (N1mΨ)—favoring ARCA—they achieved increased transcriptional yield, improved protein translation, and reduced immunogenicity *in vitro* 25. This protocol provides a more accessible and cost-effective method for mRNA production in both basic and translational research 24, 26.

#### 2.1.6 Immunogenicity

Exogenous mRNA can trigger immune responses *in vivo* by being recognized by pattern recognition receptors (PRRs) located on the cell surface, within endosomes, or in the cytoplasm 27. The immunogenicity of mRNA has both beneficial and detrimental effects. On the one hand, it facilitates the maturation of dendritic cells and activates T and B lymphocytes, resulting in strong adaptive immune responses. On the other hand, excessive activation of innate immunity can suppress antigen expression and impair overall vaccine efficacy. Therefore, careful evaluation and modulation of mRNA immunogenicity are essential for the successful design of mRNA vaccines.

#### 2.1.7 AI-guided mRNA design

Due to its single-stranded nature, mRNA is inherently unstable and prone to degradation, which can compromise its immunogenicity and reduce efficacy during storage, transport, and *in vivo* expression 28, 29. Enhancing mRNA stability has thus become a major challenge in the development of mRNA vaccines. Studies have shown that increasing the secondary structure of the mRNA sequence improves both its stability and translational efficiency 30, 31. Consequently, mRNA design algorithms must be capable of simultaneously optimizing structural stability and codon usage to enhance the performance of mRNA-based vaccines and therapeutics 32-34. To address this need, researchers developed an algorithm called LinearDesign, which jointly optimizes mRNA secondary structure and codon composition. Experimental validation demonstrated that LinearDesign significantly improved mRNA half-life and protein expression *in vitro*, and enhanced antibody responses *in vivo* by up to 128-fold compared to conventional codon optimization strategies 35. In addition to structural features, efficient translation is also influenced by the 5′ untranslated region (5′ UTR), which plays a critical role in determining mRNA vaccine potency. However, chemical modifications such as N1-methyl-pseudouridine (m1Ψ) can alter 5′ UTR function. Zhu *et al.* 36 applied artificial intelligence to design nanostructured mRNA vaccines. By leveraging machine learning on datasets from various nanoparticle delivery systems, they created platforms capable of efficiently co-delivering mRNA antigens and cyclic GMP-AMP (cGAMP) to target tissues, thereby boosting immune responses while minimizing off-target immunogenicity. Given the complex interplay between RNA design, manufacturing processes, and delivery strategies, a comprehensive understanding of their impact on clinical immunogenicity and safety is essential. Integrating computational modeling and quantitative systems pharmacology into clinical trial design enables early hypothesis generation—such as identifying optimal dosing regimens or predictive immunological biomarkers—based on preclinical and early-phase clinical data, even prior to the availability of complete human trial outcomes.

### 2.2 *In vivo* delivery of mRNA therapeutics

Synthetic mRNA provides a safe and efficient platform for gene delivery in the adult heart. Compared with viral vectors, mRNA enables transient and controllable gene expression, making it a safer alternative for delivering cardiac regenerative factors. For instance, although adenoviral delivery of stem cell factors initially supported cardiac repair, it eventually led to the development of cardiac rhabdomyosarcoma 37. In contrast, mRNA's post-transcriptional mechanism bypasses the need for nuclear entry, allowing direct cytoplasmic translation. Furthermore, mRNA delivery permits the co-expression of multiple genes in defined ratios, enabling personalized therapies based on disease stage. Xiao *et al.* 37 demonstrated that synthetic mRNA encoding STEMIN and YAP5SA efficiently transfected adult cardiomyocytes both *in vitro* and *in vivo*, resulting in improved cardiac function and reduced myocardial fibrosis in infarcted mouse left ventricles.

Despite its therapeutic potential, a major challenge in mRNA therapy is achieving efficient delivery and cellular uptake 26. As a large, negatively charged molecule, mRNA cannot freely cross the cell membrane, resulting in poor uptake when administered in its naked form. Consequently, effective intracellular delivery remains a significant barrier to therapeutic success. To overcome this, mRNA is typically encapsulated in synthetic particles composed of biocompatible materials. These carriers protect mRNA from enzymatic degradation and facilitate cytosolic release. Additionally, ligand modification of delivery systems can enable targeted delivery to specific cell types during systemic administration.

To achieve effective cardiac gene therapy, delivery platforms must satisfy several essential criteria: efficient uptake by cardiomyocytes, controlled expression kinetics, minimal immune activation, high translational efficiency and stability in cardiac tissue, and use of biocompatible carriers that do not interfere with gene function 26. Delivery platforms such as lipid nanoparticles 38, 39, polymeric particles 40, dendrimers 41, and biomimetic nanoparticles 42 have demonstrated strong potential in protecting mRNA and enhancing its uptake by target cells.

#### 2.2.1 Lipid nanoparticles (LNPs)

Lipid nanoparticles (LNPs) represent one of the most advanced platforms for RNA delivery, offering protection against RNase-mediated degradation and facilitating efficient cellular uptake. Their clinical relevance was first demonstrated in 2018 with the approval of Onpattro®, a double-stranded siRNA therapy for hereditary transthyretin-mediated (hATTR) amyloidosis 43-45. More recently, during the COVID-19 pandemic, LNPs gained widespread recognition as essential carriers for mRNA vaccines, which were rapidly developed and safely administered to millions of people worldwide 46-48.

To optimize cardiac mRNA delivery, researchers 49, 50 have evaluated various LNP formulations in citrate buffer using state-of-the-art mRNA constructs. The C12-200 LNP formulation was initially selected based on its proven effectiveness in preclinical siRNA and mRNA studies. Modifying the helper lipid (DOPE or DOPC) or adjusting the molar ratio of C12-200 did not significantly alter key physicochemical parameters such as particle size, surface charge, polydispersity, or mRNA encapsulation efficiency. *In vitro*, enhanced transfection efficiency was observed in epicardial cells and iPSC-derived fibroblasts treated with C12-200 (40%) combined with cholesterol and DOPE (15%). However, 24 hours after intramyocardial injection, no significant differences in biodistribution or cardiomyocyte targeting were observed. Furthermore, outcomes using lipofectamine 2000-complexed luciferase mRNA *in vivo* did not correlate with *in vitro* findings, highlighting a common limitation: *in vitro* delivery efficiency often fails to predict *in vivo* performance due to the complex physiological environment, including tissue structure and biodistribution dynamics 51.

To address these challenges, Sultana *et al.* 52 achieved successful intramyocardial delivery of 100 µg of mRNA via three injections into the left ventricular wall in mice. Using a similar strategy, Magadum *et al.* 53-56 demonstrated therapeutic effects in heart failure models using mRNA constructs encoding Pip4k2c, Pkm2, or FSTL1.

Among available lipid-based transfection reagents, RNAiMAX has shown robust and stable transfection efficiency *in vitro* in human induced pluripotent stem cell-derived cardiomyocytes (hiPSC-CMs). Other reagents such as TransIT (<90%), JetMESSENGER (<90%), and MessengerMax (<80%) have also enabled stable delivery of modified mRNA (m1Ψ) in cardiomyocytes 52.

*In vivo*, Zangi *et al.* 57 delivered mRNA encoding VEGFa, β-galactosidase, or luciferase into the myocardium using RNAiMAX, achieving sustained mRNA (m1Ψ) translation for up to one week and facilitating therapeutic benefits after MI. Similarly, Huang *et al.* 58 used RNAiMAX to deliver IGF1 into infarcted mouse hearts, demonstrating cardioprotective effects. However, increased apoptosis was observed near the injection site, underscoring the need for safe and precisely targeted delivery approaches for cardiac applications.

To improve delivery specificity and translational efficiency, Turnbull *et al.* 59, 60 developed functionalized lipid nanoparticles (FLNPs) and evaluated the delivery of chemically modified mRNA (m1Ψ + m5C). Their study demonstrated that FLNP-delivered GFP mRNA was successfully translated into protein within 20 minutes in the myocardium of both rats and pigs. Building upon these findings, the EPICCURE clinical trial became the first to assess the feasibility and safety of direct intramyocardial mRNA injection in patients undergoing elective coronary artery bypass grafting 26. Despite the small sample size, the study confirmed the safety and tolerability of this delivery approach 55, 61. Despite these advances, naked mRNA remains inherently unstable under physiological conditions and suffers from poor cellular uptake and inefficient endosomal escape. As a result, high doses are typically required to achieve therapeutic protein expression, which increases the risk of immune activation. In contrast, LNP-mediated delivery enables significant dose reduction, improving both safety and translational efficiency. Supporting this, Turnbull *et al.* demonstrated that relatively low doses of LNP-formulated mRNA were sufficient to induce cardiac protein expression in both small and large animal models 60.

The clinical success of mRNA-LNP-based COVID-19 vaccines has prompted broader interest in LNPs for other therapeutic applications, including targeted tissue regeneration. Cardiac nucleic acid delivery represents a particularly promising approach for myocardial repair. Optimizing LNP composition for heart-specific delivery could open new therapeutic frontiers in cardiovascular medicine. However, off-target delivery—particularly to the liver and spleen—remains a key limitation. Continued refinement of LNP design is therefore essential to minimize immunogenicity and enhance organ-specific delivery 54.

#### 2.2.2 Polymeric nanoparticles

Polymeric nanoparticles were initially developed in combination with polyethyleneimine (PEI) for nucleic acid delivery. These carriers enhance mRNA stability, allow for sustained release, and help reduce immune activation. Studies have shown that PEI-mediated delivery of RNA targeting leukocyte recruitment-related genes can suppress inflammation and preserve cardiac function in murine models of MI. However, a major limitation of polymeric nanoparticles is their cytotoxicity. For instance, PEI has been reported to disrupt cell membrane integrity and activate mitochondria-mediated apoptosis in human cell lines. Therefore, further research is needed to determine safe and effective dosage levels for polymer-based vectors. Rodness *et al.* 62 used chitosan-encapsulated VEGF nanoparticles for intramyocardial delivery in rats with myocardial ischemia. The treatment enhanced angiogenesis and promoted cardiac tissue repair while preventing the onset of heart failure. Iron oxide nanoparticles, noted for their high biocompatibility and superparamagnetic properties, offer additional benefits by enabling controlled localization and aggregation under external magnetic fields 63. These properties allow for targeted and tunable vaccine release, enhancing both efficacy and safety. In addition, iron oxide functions as an effective adjuvant by promoting the polarization of proinflammatory macrophages, enhancing immune cell activation, and stimulating cytokine production 64. However, excessive generation of reactive oxygen species (ROS) by iron oxide nanoparticles can damage DNA, proteins, and membrane lipids, potentially harming healthy cells 65. Therefore, minimizing the cytotoxic effects of polymeric nanoparticles remains a critical barrier to their clinical application.

#### 2.2.3 Peptide and protein-based nanoparticles

Peptide- and protein-based bionanomaterials have been extensively employed in drug delivery, disease diagnostics, and vaccine development due to their excellent biocompatibility and ease of production. Among them, protein nanoparticles—typically ranging from 20 to 200 nm in diameter—are particularly suitable for targeting lymph nodes. One effective and safe strategy for vaccine delivery involves the use of self-assembling protein carriers. Chen *et al.* 66 developed P-MSN/miR199a-5p nanoparticles using the CSTSMLKAC peptide. Intravenous administration of these nanoparticles improved myocardial contractility and reduced apoptosis, demonstrating therapeutic potential in MI repair. In another study, Douglas *et al.* 67 engineered a protein-lipid vehicle (PLV) by incorporating fusion-associated small transmembrane (FAST) proteins—derived from fusogenic reoviruses—into a biocompatible lipid formulation. The results showed that FAST-PLVs can efficiently deliver mRNA or DNA vaccines while avoiding the immunogenicity and toxicity commonly associated with cationic lipid nanoparticles, as well as the necrosis and tissue damage often observed with electroporation. Overall, protein-based carriers offer a promising platform for the design and development of next-generation mRNA vaccines.

#### 2.2.4 Dendrimers

Dendrimers 68 are synthetic polymers with highly branched, well-defined structures and multivalent surface functionalities, making them attractive candidates for mRNA delivery. In cardiac applications 69, coronary infusion of dendrimers carrying the β-galactosidase gene has been shown to support protein expression in cardiomyocytes for 7 to 14 days. Likewise, intramyocardial injection of dendrimers loaded with the relaxin gene in MI rat models improved cardiac function 70. However, prolonged gene delivery was associated with adverse effects, including impaired cardiac repair, highlighting a potential safety concern. Collectively, these findings underscore the potential of dendrimers for targeted mRNA delivery to the heart, while also emphasizing the need for further investigation into their long-term safety and optimal dosing strategies.

#### 2.2.5 Biomimetic nanoparticles

Biomimetic nanoparticles are created by integrating biological materials onto the surface of synthetic nanoparticles, enabling them to replicate the structural and functional properties of native cells 71. Among these, extracellular vesicles (EVs)—nanoscale vesicles enclosed by lipid bilayers—are considered “natural lipid nanoparticles.” Michael E. *et al.* 72 developed EV-like vesicles (ELVs) and loaded them with miR-126. Their study demonstrated that miR-126-loaded ELVs significantly reduced infarct size, fibrosis, and cardiomyocyte hypertrophy following MI. Building on this, Wang *et al.* 73 engineered targeted EVs (TeEVs) by attaching a cardiac-targeting peptide to miR-222-loaded EVs. These TeEVs were encapsulated in a mechanically engineered hydrogel to form an injectable cardiac patch, allowing for minimally invasive delivery. The TeEVs precisely targeted the injured myocardium and promoted the survival of damaged cardiomyocytes. Continuous delivery of TeEVs to the infarcted area alleviated ischemia-reperfusion injury and reduced ventricular remodeling.

Together, these findings underscore the enhanced biological activity and therapeutic efficacy of modified EVs. In addition to natural EVs, extracellular vesicle mimics (EVMs) 74—produced through physical or chemical disruption of cells—offer further advantages. EVMs can be manufactured at yields hundreds of times greater than natural EVs through methods such as direct mechanical fragmentation, removal of cytoplasmic and nuclear contents, or fusion of liposomes with secreted EVs. The method of preparation significantly influences EVM surface markers and internal cargo composition, allowing researchers to tailor them for specific biological functions, therapeutic applications, or drug delivery strategies.

## 3. Routes of Administration for mRNA Formulations in Cardiovascular Diseases

In addition to carrier-related properties, such as particle size, surface charge, and hydrophilicity, the route of administration plays a crucial role in determining the biodistribution and therapeutic efficacy of mRNA 75. Intravenous and intramuscular injections remain the most widely used methods for delivering polynucleotides to the heart 76. Subcutaneous and intravenous routes are also effective for targeting biomolecules in the bloodstream. For example, antisense oligonucleotides (ASOs), which are used to reduce blood lipoprotein levels, are typically administered subcutaneously. However, subcutaneous and intramuscular injections present several limitations, such as the risk of needle-induced injury, limited vaccine stability, the need for skilled healthcare professionals, and increased logistical costs due to cold-chain storage and transportation 77, 78. To address these challenges, researchers have explored transdermal drug delivery systems as non-invasive and painless alternatives to oral or injectable methods 75, 76. Despite these advances, the stratum corneum, the outermost layer of the skin, poses a significant barrier to the transdermal delivery of high-molecular-weight biomolecules like mRNA 79. To overcome this challenge, several enhancement techniques have been developed, including ultrasound 80, thermal ablation 81, electroporation 82, and microneedle (MN) systems 83. Among these, MN technology has garnered particular attention. MNs enable localized and controlled drug delivery, maintaining therapeutic concentrations over extended periods, and can be engineered to deliver mRNA directly into the dermis for targeted therapeutic action 84, 85. Moreover, MNs offer advantages in scalability and logistics, such as lower manufacturing costs and the potential to eliminate cold-chain dependencies. These benefits position microneedles as a promising strategy for both vaccine administration and broader mRNA-based therapeutic applications 86.

Currently, intramyocardial and intracoronary injections are the primary strategies for cardiac-specific drug delivery 87. Intracoronary injection involves delivering polynucleotides via a catheter inserted into the coronary artery, while intramyocardial injection requires direct catheter insertion into the heart muscle. Although both methods are effective, their invasive nature poses risks of serious complications, such as MI and even death. While the incidence of major adverse events remains low, catheter-based procedures increase the risk of complications. To address these limitations, hydrogels have emerged as a promising alternative for localized mRNA delivery. These biomaterials can be implanted into the heart via surgical or interventional methods, providing sustained, site-specific release of mRNA. This prolonged local delivery enhances the biological activity of the therapeutic agent and optimizes its efficacy in cardiac tissue.

Aerosolized drug delivery for cardiovascular diseases remains in the exploratory phase, but its potential is receiving increasing attention. The lungs, with their extensive capillary network, allow for rapid drug absorption through the alveoli. This route bypasses the hepatic first-pass effect, potentially offering faster onset of action compared to oral administration. Liu *et al.* 88 developed inhalable exosomes loaded with IL-12 mRNA (IL-12-Exo) and demonstrated that IL-12-Exo enhanced IFNγ-mediated immune activation, systemic immunity, and immunological memory in a murine lung cancer model, resulting in effective tumor suppression and prevention of recurrence. In a related study 88, the same team introduced a charge-assisted stabilization (CAS) strategy, which induces electrostatic repulsion between lipid nanoparticles (LNPs) to improve colloidal stability. By optimizing surface charge using peptide-lipid conjugates, CAS-LNPs maintained high stability during nebulization and enabled efficient pulmonary mRNA delivery in mice, dogs, and pigs.

**Figure 6:**
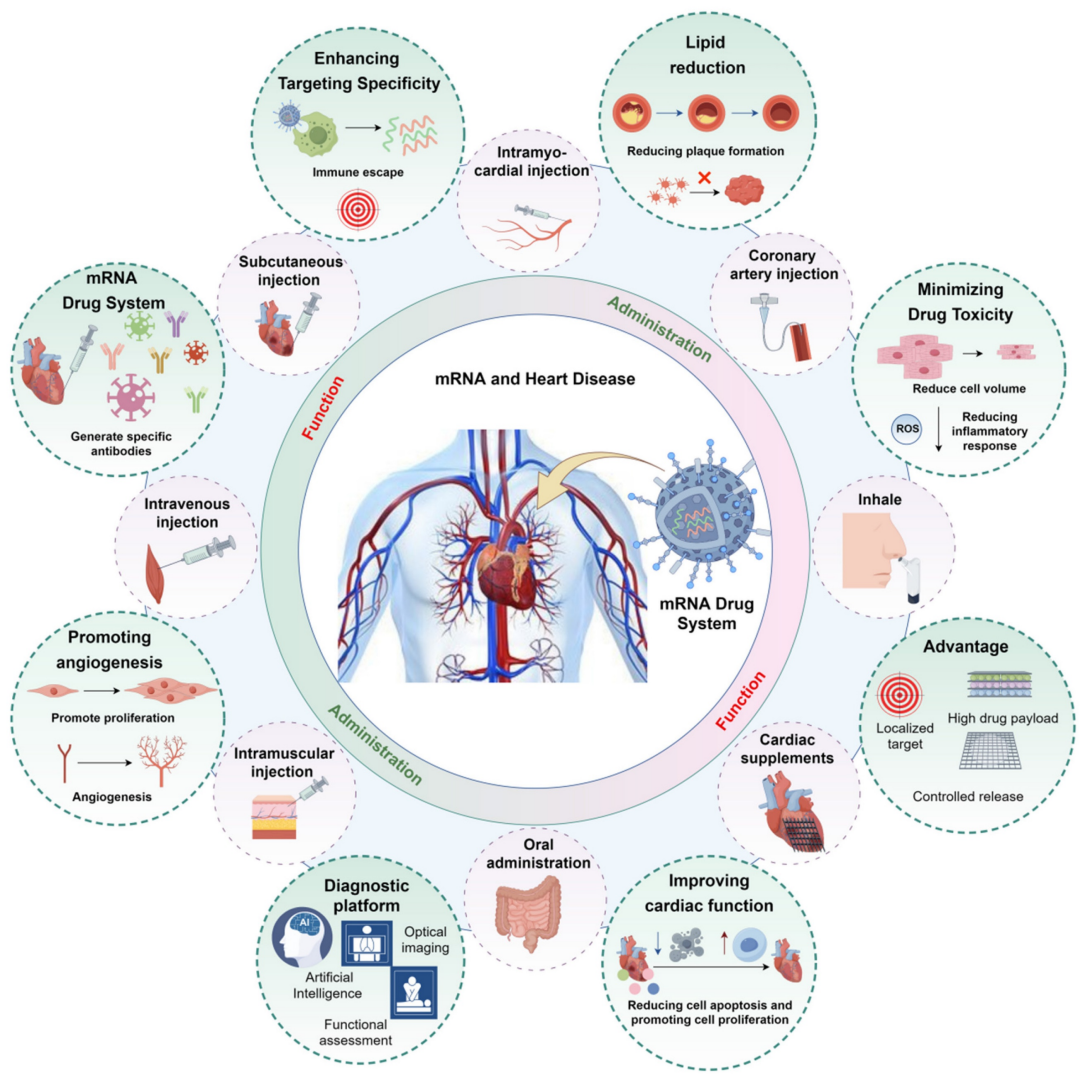
** Application of mRNA drug system in cardiovascular diseases (by Figdraw).** Based on the varying mRNA therapeutic formulations, different administration routes should be selected, such as: inhalation, intravenous, oral, intramuscular injection, subcutaneous injection, intramyocardial injection, cardiac patch, and intracoronary administration (The red picture is the mode of administration of mRNA drugs in cardiovascular diseases). After targeting the heart, mRNA drugs can improve cardiomyocyte function, promote angiogenesis, reduce inflammatory responses, and decrease plaque formation, thereby improving cardiac function in damaged hearts. mRNA is also expected to become a means of diagnosis of cardiovascular system diseases (The green picture shows the biological role of mRNA drugs in cardiovascular diseases).

These findings suggest that aerosolized delivery can effectively target pulmonary tissues and potentially facilitate secondary drug distribution to the heart through the circulatory system, offering a promising strategy for future heart-targeted therapies. In clinical settings, nebulized furosemide has been experimentally used in emergency departments to treat acute pulmonary edema, often combined with intravenous therapies to accelerate symptom relief in heart failure. Inhaled prostacyclins, such as iloprost, have also been used to reduce pulmonary arterial pressure in patients with pulmonary hypertension 89. Collectively, these studies provide a theoretical foundation for the application of inhaled therapeutics in cardiovascular care. However, current clinical applications are still limited to specific scenarios, such as emergency administration of nitroglycerin 90, or are under investigation in clinical trials. At present, inhalation-based therapies should only be administered under medical supervision and are not a substitute for conventional methods. Nevertheless, with continued advancements in delivery systems and precision-targeting technologies, aerosolized mRNA delivery may represent a new frontier in cardiovascular therapeutics. Therefore, selecting the appropriate administration route for each mRNA formulation is essential to optimize drug retention and therapeutic outcomes** (see Figure 6)**.

## 4. Application of mRNA Formulations in Cardiovascular Diseases

Inflammation plays a critical role in the development and progression of cardiovascular diseases 91. As a result, many researchers are focusing on modulating inflammation to reduce the risk of these conditions. However, traditional anti-inflammatory strategies have shown limited effectiveness in cardiovascular disease treatment. Upon administration, mRNA formulations can trigger complex immune responses, engaging both innate and adaptive immunity, potentially improving cardiovascular health through immunomodulation. Despite this potential, research in this area remains limited. In 2020, the phase III clinical trial of the small interfering RNA (siRNA) drug, inclisiran, demonstrated its efficacy and safety, showing potential benefits for cardiovascular patients by reducing plasma levels of low-density lipoprotein cholesterol (LDL-C) 92. As a "close relative" of siRNA drugs, the application of mRNA vaccines in cardiovascular diseases raises important questions about their potential, which warrants further investigation** (Figure 6)**.** Table 1** details the application of mRNA technology in cardiovascular diseases.

### 4.1 Atherosclerosis

Extensive evidence from human genetics, basic research, and clinical epidemiology has established low-density lipoprotein cholesterol (LDL-C) as a central pathogenic driver of atherosclerosis 93, 94. Meta-analyses of randomized clinical trials further support a linear association between absolute LDL-C reduction and decreased cardiovascular events across diverse patient populations 95, 96. Because the risk and progression of atherosclerotic cardiovascular disease (ASCVD) are directly proportional to cumulative LDL-C exposure over time (i.e., LDL-years), sustained and effective LDL-C lowering is critical to improving clinical outcomes 97. In line with this evidence, current clinical guidelines recommend both lifestyle modifications and pharmacologic interventions to reduce LDL-C levels, with particular focus on two high-risk groups: individuals with familial hypercholesterolemia and those with established ASCVD 98, 99. However, despite strong supporting data and multiple approved lipid-lowering therapies, most high-risk patients still fail to achieve target LDL-C levels under current chronic care models. For instance, registry data indicate that only 22% of U.S. patients and 2.7% of patients globally with heterozygous familial hypercholesterolemia attain LDL-C levels below 70 mg/dL. Similarly, only 11% to 27% of patients with ASCVD meet guideline-recommended targets 100-102. One major contributing factor is the burden imposed by chronic treatment regimens, which often require daily oral medications or periodic injections. Achieving therapeutic goals typically relies on multiple factors, including the clinician's willingness to intensify treatment 101, 102, consistent insurance coverage 103, 104, drug affordability 105, and routine access to lipid monitoring 106—all of which are frequently inadequate 107. To address these limitations, novel therapeutic paradigms such as one-time treatments are being explored. These approaches may offer more sustainable solutions for achieving long-term LDL-C control in patients with familial hypercholesterolemia and ASCVD 97.

In this context, *in vivo* gene editing has emerged as a promising therapeutic strategy that enables the permanent modification of disease-associated genes within specific organs, such as the liver 108. This approach is particularly relevant to ASCVD, as natural loss-of-function mutations in the PCSK9 gene are associated with lifelong reductions in LDL-C, favorable safety profiles, and significantly lower ASCVD risk 109, 110. These observations have driven the development of gene-editing therapies targeting PCSK9. Pharmacologic inhibition of PCSK9 (proprotein convertase subtilisin/kexin type 9) has further validated the therapeutic relevance of this pathway 92, 111-114. Multiple preclinical studies using gene-editing tools—including nucleases and CRISPR base editors—have demonstrated successful PCSK9 disruption in the livers of non-human primates 115-117. Foundational research supporting the development of VERVE-101, a CRISPR-based therapy, has involved primary human cells, murine models, and four non-human primate species, with follow-up durations extending up to eight months after treatment 115. Despite these encouraging results, several critical concerns remain. These include the long-term durability of gene editing, potential off-target effects in non-hepatic tissues, and the possibility of unintended germline modifications 94.

Building on this foundation, VERVE-101 is an investigational CRISPR base-editing therapy that consists of mRNA encoding an adenine base editor and guide RNA targeting the PCSK9 gene. These components are encapsulated in lipid nanoparticles (LNPs) and delivered via a single intravenous infusion. In parallel, researchers 115 developed an epigenetic editor specifically targeting human PCSK9. In preclinical studies involving 36 non-human primates monitored for at least one year, as well as germline editing assessments in sexually mature male primates and female mice, a single dose of the LNP-encapsulated epigenetic editor achieved near-complete silencing of PCSK9 expression in transgenic mice 94. This silencing effect persisted for at least one year and was maintained even after liver regeneration following partial hepatectomy. Notably, the gene silencing was reversible upon treatment with targeted epigenetic activators that demethylate the PCSK9 locus. In crab-eating macaques, a single administration of the epigenetic editor reduced circulating PCSK9 protein levels by approximately 90% and LDL-C levels by 70%, with durable effects 118.

These findings underscore the broader therapeutic potential of *in vivo* gene and epigenetic editing for cardiovascular risk reduction. Supporting this concept, a phase II clinical trial of the antisense oligonucleotide AKCEA-APO(a)-L—designed to target LPA mRNA—successfully reduced lipoprotein(a) [Lp(a)] concentrations to below 50 mg/dL, a clinically safe threshold, in over 90% of treated patients 26. Lp(a), encoded by the LPA gene, is a genetically determined and independent risk factor for cardiovascular disease. Elevated Lp(a) levels are associated with increased cardiovascular risk and are largely unresponsive to conventional lipid-lowering therapies. Most Lp(a)-lowering treatments currently in development require repeated dosing. In contrast, gene-editing strategies offer the promise of a single-dose, long-lasting solution 119.

To this end, Ramon and colleagues developed a gene-editing approach using mRNA encoding transcription activator-like effector nucleases (TALENs) to disrupt the LPA gene. *In vitro* screening identified TALEN constructs capable of precise gene editing with minimal off-target effects. The TALEN mRNA was subsequently encapsulated in a proprietary lipid nanoparticle platform (LUNAR) and administered to transgenic mice expressing human LPA. A single injection resulted in an over 80% reduction in plasma Lp(a) levels, with sustained effects lasting at least five weeks. These findings highlight the feasibility of gene-editing-based therapies to reduce cardiovascular risk and offer a potential long-term treatment for patients with elevated Lp(a) 119.

In parallel with genetic approaches, recent attention has turned to immune-mediated mechanisms of plaque destabilization, which represent another promising avenue for therapeutic intervention in atherosclerosis 120. An increasing accumulation of bone marrow-derived cells and lymphocytes within atherosclerotic plaques has been associated with fibrous cap thinning and plaque destabilization—key features of vulnerable plaques that are prone to rupture and can lead to thromboembolic events. Mechanistically, activated CD4⁺ T cells and elevated levels of interferon-γ (IFN-γ) suppress collagen synthesis, compromising the structural integrity of the fibrous cap. Simultaneously, activated macrophages secrete cathepsins that degrade collagen and elastin, further weakening plaque stability 121. Among immune cell subsets, Th1 cells play a particularly important role in promoting atherogenesis by producing pro-inflammatory cytokines such as IFN-γ, TNF-α, and IL-2. These cytokines amplify the inflammatory cascade by activating macrophages and additional T cells, thereby intensifying plaque inflammation 122.

mRNA vaccines may help counteract these pro-atherogenic processes. By suppressing Th1 cell activity, reducing the secretion of inflammatory cytokines (e.g., IFN-γ), and disrupting Th1-macrophage interactions, these vaccines may reduce local immune activation. Collectively, these actions could attenuate inflammation within plaques and enhance fibrous cap stability. Overall, mRNA vaccines represent a promising therapeutic strategy for both the prevention and treatment of atherosclerosis. They offer the potential to inhibit the formation of new plaques and stabilize existing ones, thereby reducing the risk of clinical complications such as coronary embolism.

### 4.2 Ischemic heart disease

Acute myocardial ischemia initiates a pro-inflammatory response aimed at removing necrotic cellular debris from the infarcted myocardium. However, myocardial reperfusion following percutaneous coronary intervention often amplifies this inflammatory cascade, leading to additional cardiomyocyte death and tissue injury, typically within 6 to 24 hours post-reperfusion. This initial inflammatory phase is followed by an anti-inflammatory reparative phase that promotes wound healing and scar formation, thereby preventing cardiac rupture. The transition between these phases is tightly regulated by complex interactions between cardiac-resident cells (such as cardiomyocytes, endothelial cells, fibroblasts, and stromal cells) and immune cells (including neutrophils, monocytes, macrophages, dendritic cells, and lymphocytes) 123. Considering the harmful effects of an exaggerated and prolonged pro-inflammatory response and the beneficial role of the subsequent reparative phase, therapeutic strategies that suppress early inflammation while promoting tissue repair may help reduce infarct size and prevent adverse left ventricular remodeling.

Over a decade of research by Kenneth Chien's team at the Karolinska Institute has shown that chemically modified mRNA encoding vascular endothelial growth factor (VEGF) can induce stable yet transient VEGF protein expression within days 124-126. This transient expression minimizes the risk of toxicity associated with prolonged VEGF exposure. In a phase 2a clinical trial involving patients undergoing cardiac surgery, direct intramyocardial injection of VEGF-mRNA was shown to be safe and improved cardiac function. These findings highlight the therapeutic potential of mRNA-based approaches for tissue repair, particularly in cardiac regeneration.

Beyond angiogenesis, immunomodulation has also emerged as a promising therapeutic avenue. Studies 127 have demonstrated that CD4⁺ T lymphocyte infiltration exacerbates early reperfusion injury following myocardial ischemia. Mice deficient in CD4⁺ T cells exhibit significantly smaller infarcts during the acute phase 128. However, during the healing phase, these mice display impaired collagen matrix formation, greater left ventricular dilation, increased cardiac rupture, and higher mortality compared to wild-type controls. In contrast, CD4⁺CD25⁺FOXP3⁺ regulatory T cells (Tregs) exert protective effects by secreting anti-inflammatory cytokines such as TGF-β and IL-10 129. These Tregs suppress the recruitment of inflammatory cells—including neutrophils, monocytes, and CD4⁺ T cells—promote macrophage polarization from a pro-inflammatory M1 to an anti-inflammatory M2 phenotype, and inhibit fibroblast-to-myofibroblast differentiation, thereby limiting fibrotic remodeling and adverse structural changes 130-132.

Given the pivotal role of immune responses in ischemic heart disease, mRNA-based therapeutics targeting immune pathways offer considerable promise. Mays *et al.* 133 developed a chemically modified mRNA encoding FOXP3. When delivered to the lungs, it upregulated FOXP3 expression and, via an IL-10-dependent mechanism, modulated immune cell recruitment and maintained balance among Tregs, Th2, and Th17 cells, preventing asthma onset. Drawing on this concept, a similar strategy applied early after myocardial ischemia could promote Treg expansion and IL-10 secretion, attenuating pro-inflammatory responses and reducing myocardial injury.

Ischemic damage also promotes activation of antigen-presenting cells (APCs) through damage-associated molecular patterns (DAMPs) released by necrotic tissue, leading to the presentation of cardiac self-antigens and activation of T and B lymphocytes 134. Autoantibodies against cardiac troponin I (cTnI-Ab) have been detected in patients with acute MI and correlate with clinical outcomes, suggesting that ischemia can disrupt immune tolerance 135. As previously noted, nucleoside-modified mRNA vaccines reduce nonspecific inflammation during antigen presentation and induce immune tolerance to self-antigens 136. These vaccines also promote Treg expansion while inhibiting Th1 responses, offering a potential strategy to mitigate post-ischemic autoimmune injury.

Importantly, CD4⁺ T cells and other immune cells, such as M2 macrophages, also contribute to the reparative phase of ischemic injury. Since anti-inflammatory mRNA therapies might interfere with this process, it is essential to restrict their activity to the early pro-inflammatory window. This could be achieved by leveraging the transient nature of mRNA through chemical modifications that reduce its half-life and confine its action to the acute phase.

Encouragingly, efforts to translate mRNA-based therapies into clinical applications have already begun. In 2017 137, AstraZeneca and Moderna Therapeutics jointly developed AZD7970, an mRNA vaccine encoding relaxin, for the treatment of heart failure. Preclinical studies showed that liposome-modified mRNA could recruit chemotactic monocytes to sites of cardiac injury, reducing inflammation, further supporting the therapeutic potential of mRNA in cardiovascular disease.

Because the generation of new cardiomyocytes after birth is minimal, preventing cardiomyocyte death is essential for effective cardiac repair. Researchers from Harvard and the Karolinska Institute 57 showed that chemically modified mRNA, when injected into infarcted mice, promoted the differentiation of cardiac stem cells into functional cardiovascular cells rather than fibrotic tissue. Notably, mRNA administered within 48 hours of infarction significantly improved cardiac function.

These findings have been supported by complementary studies. Liu *et al.* 138 demonstrated that miR-141, delivered via tail vein injection, downregulated ICAM-1 expression in the heart, reduced serum cTnI and LDH levels, and mitigated ischemia/reperfusion injury. Sun *et al.* 139 used RGD-PEG-PLGA nanoparticles to deliver mRNA targeting the SIRT3/AMPK pathway, reducing apoptosis, inflammation, and oxidative stress. Nie *et al.* 140 developed a Hep@PGEA-based delivery system for miRNA-499, achieving cardiac-specific targeting and functional improvement. Turnbull further showed that lipid-like nanoparticles could efficiently deliver mRNA to the heart and induce transient protein expression.

Taken together, these studies underscore the promise of mRNA-based interventions for myocardial repair. When applied prophylactically in high-risk populations with acute coronary syndrome, such therapies may reduce the incidence of MI-related disability and mortality, ultimately improving long-term patient outcomes.

### 4.3 Myocarditis

mRNA vaccines 141, as a novel class of nucleic acid-based therapeutics, offer distinct advantages and show considerable potential in the prevention and treatment of viral myocarditis. By designing mRNA sequences that encode viral antigenic epitopes, these vaccines enable *in vivo* expression of target proteins. Such proteins serve as effective alternatives to inactivated or attenuated vaccines, as their degradation products are non-toxic amino acids, and their structurally defined, purified antigens effectively induce both cellular and humoral immune responses. Unlike DNA vaccines, mRNA is translated directly in the cytoplasm without needing to cross the nuclear membrane, resulting in greater intracellular flexibility, higher translational efficiency, and reduced safety risks—such as insertional mutagenesis 142. Additionally, naturally occurring RNA sequences from coxsackievirus B3 (CVB3) may serve as templates for mRNA vaccine design, supporting scalable production 143.

Beyond general immunogenic benefits, mRNA vaccines have shown particular advantages in minimizing antigenic cross-reactivity. For example, a 2017 study published in Cell by Richner *et al.* 143 described a modified mRNA vaccine encoding a mutated prM-E gene of Zika virus (ZIKV). By disrupting a conserved fusion loop epitope in the E protein, the vaccine protected mice from ZIKV infection while reducing cross-reactive antibodies that could otherwise enhance dengue virus infection. A similar strategy applied to CVB3 could potentially elicit robust neutralizing antibody responses to control viral replication and inflammation, while also reducing autoimmune activation and myocardial injury.

Although the pathogenesis of viral myocarditis remains incompletely understood, it is most frequently associated with infections caused by CVB3, adenoviruses, echoviruses, and more recently, SARS-CoV-2 144. Several preclinical studies have explored CVB3-targeted vaccination strategies, including inactivated, live attenuated, DNA, and virus-like particle (VLP) vaccines. For instance, Park *et al.* 145 developed a highly attenuated CVB3 mutant (YYFF) by substituting two conserved tyrosine residues in the VP2 C-terminal region with phenylalanine, which induced high titers of neutralizing antibodies and CD8⁺ T cell responses in mice. Kim *et al.* 146 constructed recombinant plasmids encoding CVB3 VP1 and VP3 proteins, which elicited strong neutralizing antibody responses and improved survival in immunized mice. Similarly, Zhang *et al.* 147 developed a recombinant baculovirus expressing the full-length CVB3 genome, which conferred protective immunity comparable to that of attenuated vaccines.

Despite these promising findings, none of these vaccine candidates have advanced to clinical application. Major limitations include the risk of reversion to virulence or incomplete inactivation for live attenuated and inactivated vaccines, the potential for genomic integration and oncogenic transformation with DNA vaccines, and the high production costs associated with certain platforms. These barriers continue to hinder clinical translation and underscore the need for novel vaccine designs and efficient delivery systems.

Complicating vaccine development further is the role of autoimmunity in CVB3-induced myocarditis. Following infection, cardiac-resident cells—including cardiomyocytes, phagocytes, and fibroblasts—secrete pro-inflammatory cytokines such as IL-1, IL-6, TNF-α, and IL-18, initiating acute inflammation 148. As the adaptive immune system is activated, antigen-specific lymphocyte responses are triggered. B cells produce neutralizing antibodies, while T cells release cytokines such as IFN-γ to suppress viral replication 149.

However, certain regions of the CVB3 proteome share sequence similarity with cardiac self-antigens, leading to molecular mimicry that activates cross-reactive T cells and promotes autoantibody production 150. Moreover, the cytolytic nature of CVB3 can result in the release of intracellular and membrane-bound cardiac antigens, further driving the activation of autoreactive lymphocytes and antibodies. This dysregulated immune response may underlie the progression from acute to chronic myocarditis and eventually to dilated cardiomyopathy, presenting additional challenges in the development of safe and effective vaccines 151.

### 4.4 Heart failure

Heart failure remains a major unmet clinical need, as current therapies provide limited benefit in halting or reversing disease progression 152. One key pathological feature of heart failure is myocardial fibrosis, driven largely by activated fibroblasts that contribute to adverse tissue remodeling. Consequently, therapeutic strategies that specifically target these pathological fibroblasts are urgently needed 152. Among emerging approaches, lipid nanoparticle (LNP)-encapsulated mRNA has shown promise. This platform, which has demonstrated clinical success in mRNA-based COVID-19 vaccines, offers a flexible and efficient means of *in vivo* protein expression. In a murine model of cardiac injury, researchers 153 utilized T cell-targeted LNPs to generate anti-fibroblast chimeric antigen receptor (CAR) T cells *in vivo*. These engineered cells not only reduced cardiac fibrosis but also migrated to fibrotic regions, where they exerted paracrine effects to suppress local inflammation. This suggests a potential immunomodulatory role in addition to direct cytotoxicity. In a separate study using a mouse model of heart disease, CD5-targeted mRNA-LNPs were employed to induce CAR T cell generation *in vivo*. The resulting T cells reduced myocardial fibrosis and restored cardiac function, further highlighting the therapeutic potential of mRNA-based cell reprogramming. Complementing these findings, another research 154 group optimized the delivery of human VEGF-A mRNA to the left ventricular myocardium in pigs. This intervention led to a marked reduction in myocardial fibrosis. Notably, intravenous and localized administration of mRNA in rodent and non-human primate models did not induce innate immune activation, supporting the safety profile of this approach. However, the invasive routes currently required for mRNA delivery may hinder the broad adoption of such therapies. To address this limitation, AstraZeneca recently developed an mRNA-LNP formulation suitable for subcutaneous administration. By incorporating a steroidal prodrug, this formulation significantly enhanced protein expression and prolonged translation duration. Such innovations are expected to improve the feasibility, scalability, and accessibility of mRNA therapeutics for treating heart failure 155.

### 4.5 Congenital heart disease

Nanoparticle-based drug delivery systems hold significant potential for advancing medical therapies 156. However, their clinical utility is often hindered by limited vascular permeability and rapid clearance by phagocytic cells. Interestingly, the fetal environment—characterized by accelerated angiogenesis, active cell proliferation, and an underdeveloped immune system—may circumvent these limitations, making in utero nanoparticle delivery a promising alternative 157. Nevertheless, current understanding of nanoparticle-mediated drug delivery during fetal development remains limited. To explore this approach, researchers 144 used Ai9 CRE reporter mice and demonstrated that lipid nanoparticle (LNP)-mRNA complexes can be delivered in utero with high efficiency and low toxicity. These complexes successfully transfected major fetal organs, including the heart, liver, kidneys, lungs, and gastrointestinal tract. At four weeks postnatally, sustained transfection was observed in 50.99% ± 5.05% of diaphragm myofibers, 36.62% ± 3.42% in the heart, and 23.7% ± 3.21% in skeletal muscle. These findings confirm that Cas9 mRNA and sgRNA delivered via LNPs can mediate genome editing in fetal organs in utero. Overall, this study establishes the feasibility of non-viral mRNA delivery to extrahepatic fetal tissues and underscores its potential as a novel therapeutic strategy for the treatment of congenital heart disease.

### 4.6 Cardiomyopathies

Gene therapy may present a potential opportunity for the treatment of various cardiomyopathies, yet alternative treatment options for some cardiomyopathies are very limited 158. Mearini *et al.* 159 have shown that a single administration of AAV9-MYBPC3 in neonatal homozygous MYBPC3-targeted knock-in mice can prevent the development of cardiac hypertrophy and lead to a dose-dependent increase in MYBPC3 protein expression. BAG3 160 is highly expressed in the heart, skeletal muscle, and central nervous system. Loss-of-function variants of BAG3 leading to decreased BAG3 levels were associated with dilated cardiomyopathy. Researchers 161 have demonstrated that intravenous injection of AAV9-BAG3 in mice can prevent the onset of dilated cardiomyopathy. Additionally, studies 162 have found that catheter-based retrograde coronary sinus infusion for the delivery of low doses of AAV9-BAG3 in healthy mini pigs can result in diffuse myocardial transduction. Furthermore, PRKAG2 163 mutations cause glycogen storage disease of the heart, characterized by a hypertrophic phenotype, supraventricular arrhythmias, and atrioventricular block. The CRISPR-Cas9 system combined with AAV9 has been used to disrupt the mutated PRKAG2 alleles encoding the H503 mutation in knock-in mice, and a single systemic injection of this product on day 4 or day 42 can restore morphology and function in the mouse model, thereby reducing left ventricular thickness and myocardial glycogen content. Additionally, treatments for cardiac amyloidosis 164 include transthyretin amyloidosis (ATTR) stabilizers that prevent tetramer dissociation and amyloid formation, such as tafamidis 165, which can reduce TTR production through RNA silencing.

Desmocollin-2 (DSC2), a desmosomal membrane-anchored protein, has long been recognized as a central pathogenic factor in arrhythmogenic right ventricular cardiomyopathy (ARVC) 166. Despite the well-established role of its mutations in disease pathogenesis, effective therapeutic strategies for DSC2 mutation-induced cardiomyopathy have remained elusive. Ge *et al.* 166 proposed a highly targeted, non-viral, and repeatable therapeutic approach by encapsulating DSC2 mRNA within lipid nanoparticles (LNPs) and administering the mRNA-based drug via echocardiography-guided intraventricular septal injection. Their findings demonstrated that a single injection of modified DSC2 mRNA effectively reversed cardiomyopathy, restored right ventricular function, and significantly prolonged survival *in vivo*. Notably, this therapeutic efficacy was observed not only in early-stage disease models using young mice but also in aged mice with advanced disease progression. Furthermore, repeated dosing in late-stage disease models reactivated therapeutic benefits and extended survival, highlighting the controllable and sustained potential of the mRNA platform. Compared to conventional gene therapies such as adeno-associated virus (AAV) vectors, this strategy enables rapid protein translation while circumventing risks associated with viral vectors, including immunogenicity and genomic integration.

Overall, these therapies have the potential to fundamentally alter the pathogenesis of diseases, but drug toxicity is dose-dependent, making the development of vectors with improved myocardial tropism and transduction efficiency crucial.

## 5. Discussion and Perspectives

mRNA-based therapeutics offer several notable advantages, including the ability to encode a wide range of proteins, induce strong immune responses, and support rapid, scalable manufacturing. These platforms also enable targeted delivery via various nanocarrier systems while minimizing systemic side effects. Moreover, because mRNA does not integrate into the host genome, it avoids the risk of insertional mutagenesis, contributing to a favorable safety profile. These attributes have sparked growing interest in applying mRNA technologies to cardiovascular diseases.

Despite these benefits, several limitations remain. A major challenge is the intrinsic instability of mRNA. Due to the widespread presence of RNases in tissues and cells, mRNA is prone to rapid degradation, compromising antigen expression and therapeutic efficacy. Another concern is the high immunogenicity of exogenous mRNA. It can be detected by multiple pattern recognition receptors (PRRs) on the cell surface and within the cytoplasm, activating innate immune pathways 167. While this immunostimulatory effect may serve as a natural adjuvant—promoting dendritic cell maturation and enhancing T and B cell responses—overactivation can result in excessive type I interferon (IFN) production, which suppresses mRNA translation and impairs antigen-specific immunity 168. In addition, the large molecular size and negative charge of naked mRNA limit its ability to cross the phospholipid bilayer of cell membranes, making intracellular delivery a critical bottleneck in the development of mRNA-based therapeutics. These limitations underscore the importance of advancing delivery systems, particularly lipid-based carriers that can protect mRNA and promote cellular uptake.

To address these challenges and enhance the clinical utility of mRNA vaccines, several molecular engineering strategies have been explored. Modifications such as locked nucleic acids (LNAs) in the 5′ cap structure can improve mRNA stability by up to 1.6-fold 169. Similarly, adding stabilizing elements to the 3′ untranslated region (3′UTR) further increases transcript half-life 170. To reduce immunogenicity, nucleoside analogs like pseudouridine (Ψ), 5-methylcytidine (m5C), and 2-thiouridine (s2U) have been incorporated to blunt Toll-like receptor (TLR) signaling and attenuate innate immune activation 15. Translation efficiency can be optimized using anti-reverse cap analogs (ARCA) and by minimizing stable secondary structures in the 5′UTR, which can impede ribosome binding 171, 172. Lipid nanoparticles (LNPs) remain the leading delivery platform due to their protective, biocompatible, and membrane-fusogenic properties 173. For example, Kranz *et al.* 174 developed mRNA-LPX, a liposomal system that successfully delivers mRNA to CD11c⁺ antigen-presenting cells in lymphoid tissues, triggering robust antigen-specific immune responses. These findings highlight the need for a systems-level understanding of how RNA design, delivery technology, and immune signaling intersect to influence clinical outcomes. Computational modeling and systems pharmacology may provide critical insights into optimal dosing, immune biomarker identification, and trial design—particularly in the preclinical and early clinical stages.

Alongside molecular optimization, the safety of mRNA-based therapeutics must be thoroughly assessed. Data from 3.644 million COVID-19 mRNA vaccine recipients (BNT162b2 and mRNA-1273), published in JAMA 175, showed that the most common adverse reactions—such as injection site pain, fatigue, headache, myalgia, and chills—generally resolved within five days and affected fewer than 10% of individuals thereafter. These results suggest an overall favorable safety profile. However, some studies have raised concerns regarding spike protein-associated cardiac toxicity, with evidence pointing to potential cardiomyocyte dysfunction and inflammation. Separately, adeno-associated virus (AAV)-mediated gene therapy in a pig model of MI led to sudden death by the seventh week post-treatment. These findings emphasize the need for rigorous safety evaluations, particularly concerning dose and route of administration, before proceeding to large-scale trials in patients with preexisting cardiac conditions.

Another critical limitation is the suboptimal therapeutic efficacy observed in many gene therapy trials targeting cardiovascular diseases. Despite six clinical trials, the gene therapy MYDICAR failed to improve cardiac function in phase II trials 176, 177. Similarly, BioBypass—an adenoviral vector encoding VEGF-121—was safe but failed to restore myocardial function in refractory ischemia patients. Other trials involving VEGF-A165, bFGF, HGF, SDF-1, and FGF4 have also demonstrated limited clinical benefit. These outcomes reflect broader translational hurdles in gene therapy for cardiovascular medicine. As such, improving efficacy while ensuring safety remains a key unmet need. Overcoming these challenges will be critical for realizing the full therapeutic potential of mRNA vaccines in cardiovascular disease.

Finally, mRNA therapy introduces a new paradigm for gene expression-based treatment that may circumvent the complications seen with DNA- and protein-based modalities. Its capacity for transient expression is particularly well-suited for regenerative applications such as myocardial repair. Nevertheless, this feature poses a barrier for long-term protein replacement therapies. If challenges related to stability, delivery, and duration of expression can be addressed, mRNA therapeutics may become a cornerstone of next-generation cardiovascular interventions—complementing existing modalities such as AAV vectors, small molecules, recombinant proteins, monoclonal antibodies, and peptide carriers 26.

## 6. Conclusion

mRNA-based formulations represent a highly promising and potentially disruptive platform for both therapeutic and preventive applications. While the scientific community eagerly awaits the first definitive clinical efficacy data, substantial opportunities remain for further development and optimization. As previously discussed, the structural configuration and cellular uptake of mRNA are key determinants of antigen expression efficiency. These factors can be strongly influenced by advances in RNA sequence design, formulation chemistry, and delivery methods. Any modifications to these parameters may significantly affect mRNA stability, translational output, or interactions with intracellular RNA sensors, and should therefore be carefully evaluated during early-stage development. For example, beyond nucleotide modifications, novel delivery strategies can greatly influence the adjuvanticity of mRNA vaccines. Although direct cytosolic delivery can enhance antigen expression, it may bypass endosomal pathways necessary for interaction with RNA-sensing receptors, potentially weakening the desired immunostimulatory response. This issue may need to be addressed through tailored design. In more challenging therapeutic scenarios, the inclusion of auxiliary mRNA molecules may serve to modulate immune responses and improve therapeutic outcomes. Furthermore, combining mRNA-based therapies with other treatment modalities may produce synergistic effects. However, such combination strategies inevitably increase the complexity of both vaccine development and clinical implementation, posing additional regulatory and manufacturing challenges. In conclusion, mRNA therapeutics offer a flexible, potent, and safe treatment platform with the potential to revolutionize cardiovascular medicine. With continued advancements in molecular design and delivery systems, mRNA technologies are well-positioned to become a central component of next-generation precision therapies.

## Figures and Tables

**Table 1:** The application of mRNA technology in cardiovascular diseases.

Disease	Carrier	Payload	Admini-stration	Therapeutic effects	Model / Phase	Refer-ences
MI	\	VEGF-A modRNA	Intramyocardial injection	Promote vascular and heart regeneration	Mouse	57
	LNP	modRNA	Intravenous infusion	Targeting of the cardiac infarct zone	Mouse	178
	RGD-PEG-PLGA NPs	mRNA	Intravenous infusion	Inhibition of cardiomyocyte apoptosis, and inflammation	Rat	139
	Hep@PGEA NPs	miRNA-499	Intravenous infusion	Targeted damage to the heart	Mouse	140
	LNP	modRNA	Intravenous infusion	Improving cardiac regeneration	Rat, Pig	60
	LNP	VEGF-mRNA	Epicardial injection	Demonstrated safety and tolerability.	Phase II clinical trial	124
	\	miR-146b	Intravenous infusion	Protect heart function	Rat	179
Atherosclerosis	NPs	m1Ψ mRNA	Intravenous infusion	Reduce the chronic inflammatory response	Mouse	136
	LNP	mRNA	Intravenous infusion	The PCSK9 levels were significantly reduced	Mouse	180
	\	ASO	Subcutaneous injection	Reduce triglycerides	Advanced clinical	181
	NPs	ABC1 mRNA	Co-culture	Reduce the cholesterol efflux	Human monocyte cell line-U937	182
	NPs	Atorvastatin	Intravenous infusion	Reduce the inflammatory response	Mouse	183
	NPs	IL-10 mRNA	Intravenous infusion	Modulate inflammation in advanced atherosclerotic lesions	Mouse	184
Heart failure	LNP	FAP mRNA	Intravenous infusion	Reduction of cardiac fibrosis	Mouse	84
	LNP	mRNA	Intravenous infusion	Reduction of cardiac fibrosis	Mouse	153
	\	mRNA-0184	Intravenous infusion	Hormone replacement therapy	Phase I clinical trial	137
	\	VEGF-A mRNA	Intramyocardial injection	Promote angiogenesis and reduce fibrosis	Pig	185
	NPs	TP-10	Inhalation	Inhibit cardiac myocyte hypertrophy and reduce fibrosis	Mouse	186
Myocardiosis	rBV	CVB3	Intravenous infusion	Anti-inflammatory action	Mouse	148
	AAV-9	MYBPC3 mRNA	Intravenous infusion	Prevent the development of cardiac hypertrophy	Mouse	159
	AAV-9	BAG3	Intravenous infusion	Prevent the development of a dilated cardiomyopathy	Mouse	163
	AAV-9	BAG3	Coronary sinus infusion	Diffuse myocardial transduction	Pig	162
	LNP	NTLA-2001	Intravenous infusion	The concentration of reducing TTR in the serum	Phase III clinical trial	187
CHD	LNP	mRNA	Intrauterine injection	Reduce cardiac toxicity	Mouse	144

Abbreviations: modRNA, modified RNA; VEGF-A, human vascular endothelial growth factor-A; NPs, nanoparticles; ASCs, Adipose-derived stem cells; EVs, Extracellular vesicles; rBV, recombinant baculovirus; CHD, Congenital Heart Disease; TTR, Transthyretin; I/R, Ischemia-reperfusion; MI, Myocardial infarction.
